# Occupational Burnout and Quality of Life Among Healthcare Professionals in Pakistan: A Cross-Sectional Analysis

**DOI:** 10.1192/j.eurpsy.2026.10748

**Published:** 2026-07-31

**Authors:** M. Ali, S. Muhammad, Z. Farooq

**Affiliations:** 1 Adult Community Mental Health Services, HSE North Dublin Mental Health Services, Dublin, Ireland; 2 Psychiatry, Pak Naval Services Shifa Hospital, Karachi, Pakistan; 3 Psychiatry, HSE Dublin North City Mental Health Services, Dublin, Ireland

## Abstract

**Introduction:**

Burnout is a growing concern among healthcare professionals in Pakistan, where socioeconomic instability, long working hours, and lack of institutional support contribute to deteriorating mental well-being. This study examines the prevalence of burnout and its relationship with quality of life (QOL) among doctors working in a tertiary care setting.

**Objectives:**

This study aimed to determine the prevalence of occupational burnout among doctors in a tertiary care hospital, evaluate its relationship with quality of life (QOL), and explore the causal pathway linking workload, exhaustion, and burnout.

**Methods:**

A cross-sectional survey of 93 healthcare professionals was conducted using purposive sampling. Participants completed the Maslach Burnout Inventory (MBI) and the World Health Organization Quality of Life- Brief Version (WHOQOL-BREF or QOL), both validated and reliable tools. Inclusion criteria required participants to be 25–65 years old, with ≥1 year of practice and current hospital employment. Burnout was assessed across occupational exhaustion (OE), depersonalization (DP), and personal accomplishment (PA), while QOL was measured in five domains. Spearman correlation was used to test associations.

**Results:**

The sample included female doctors (64.5%) and male doctors (35.5%), most of whom were postgraduate trainees (68.8%) aged 24–30 years (58.1%). A majority worked ≤80 hours/week (86%) and earned <1.2 million PKR annually (83.9%). Burnout was highly prevalent across both genders: 79.6% reported moderate to high OE, 81.7% high DP, and 67.7% low PA. Importantly, PA functioned as a protective factor, showing a positive association with psychosocial well-being (r = 0.209, p = 0.045). Overall QOL scores were moderate, with deficits most evident in psychosocial and social domains. OE demonstrated strong negative correlations with overall QOL (r = –0.459), physical health (r = –0.557), and psychosocial health (r = –0.414), all p < 0.001, while DP was weakly but significantly linked to lower QOL and environmental satisfaction.

**Image 1:**
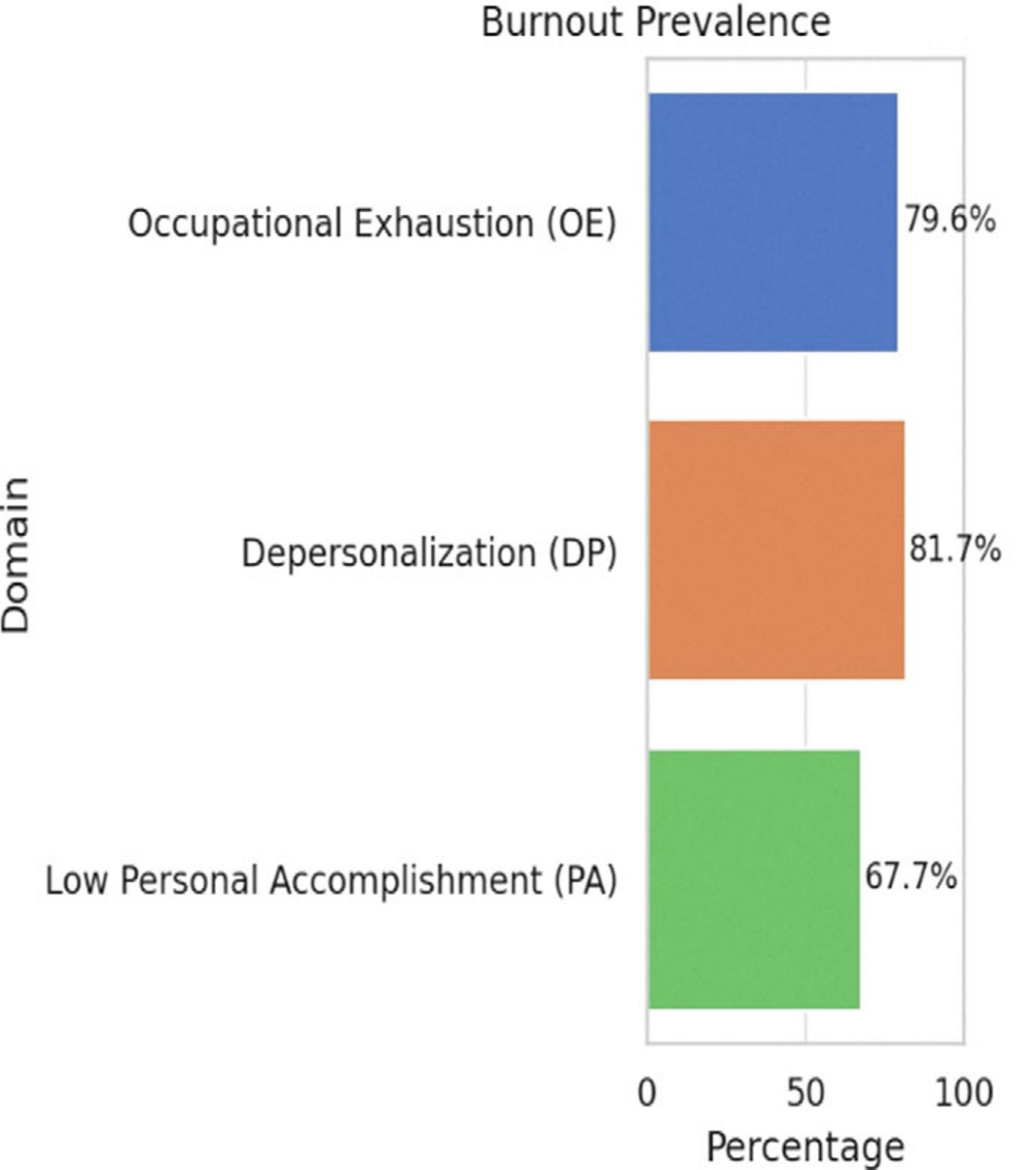

**Image 2:**
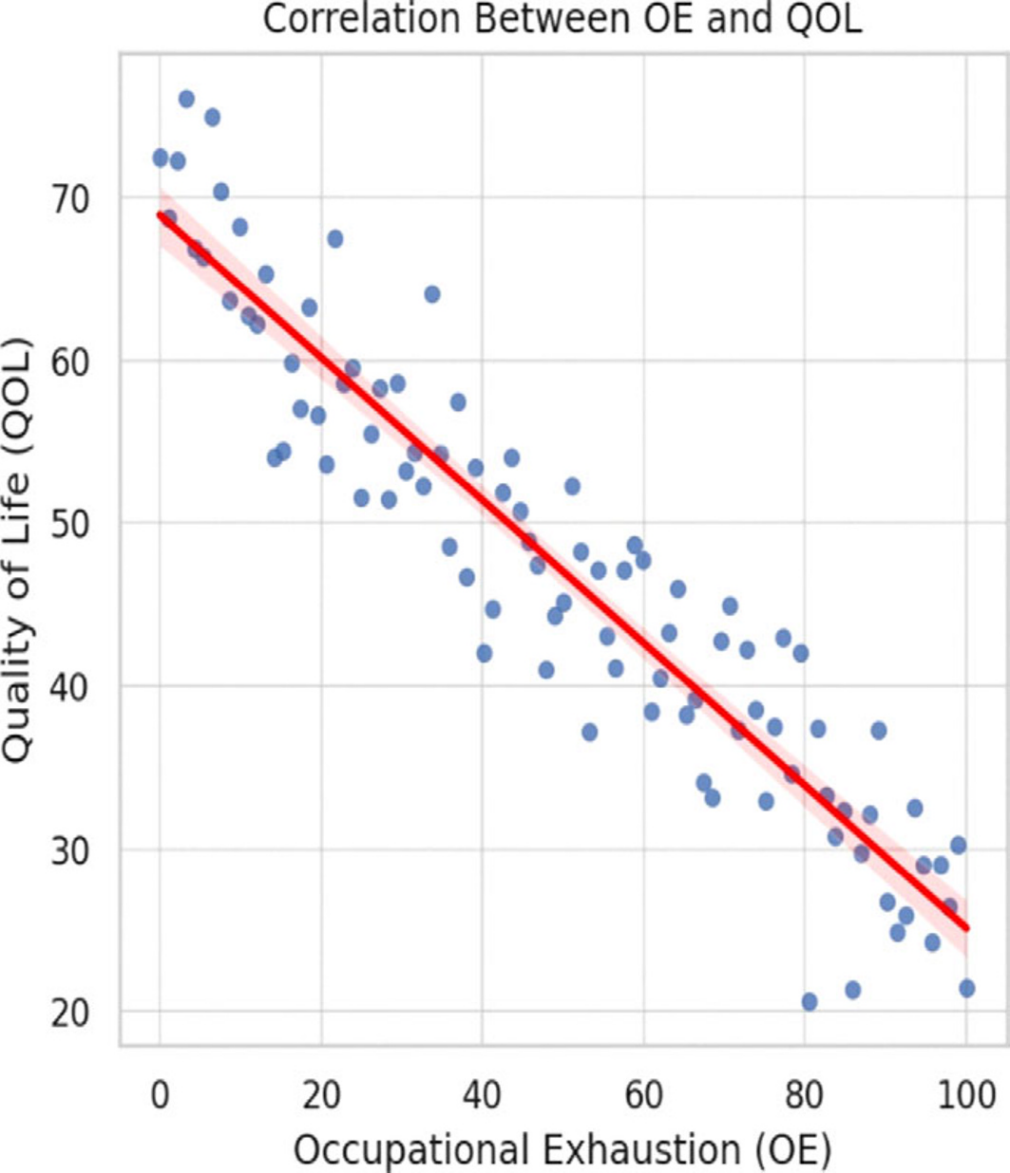
Image 2: Long description.

**Conclusions:**

Burnout is alarmingly common among both male and female young doctors in Pakistan, with trainee doctors particularly affected. Findings support a causal pathway wherein heavy workload drives occupational exhaustion, which in turn leads to full burnout. Personal accomplishment acts as a buffer against this trajectory. Institutional interventions—including regulated working hours, counseling services, and peer support programs, along with job security and mental health support—are urgently needed to safeguard the healthcare workforce amidst Pakistan’s systemic challenges.

**Disclosure of Interest:**

None Declared

## Long descriptions

### Image 2: Long description

The x-axis is labeled Occupational Exhaustion O E with a scale from 0 to 100. The y-axis is labeled Quality of Life Q O L with a scale from 20 to 70. A dense collection of blue circular data points is distributed across the plot. A solid red regression line starts at approximately 69 on the y-axis when x is 0 and descends with a constant negative slope to approximately 25 on the y-axis when x is 100. A light red shaded area surrounds the regression line representing the confidence interval. The data points are tightly clustered around this line, indicating a strong negative correlation where higher levels of O E correspond to lower levels of Q O L. There are a few outliers, notably a point near x equals 80 and y equals 20.

Navigate back to Image 2:.

